# Visitor preferences and satisfaction in Attica zoological park, Greece

**DOI:** 10.1016/j.heliyon.2020.e04935

**Published:** 2020-09-14

**Authors:** Paraskevi Karanikola, Thomas Panagopoulos, Stilianos Tampakis, Antonios Tampakis

**Affiliations:** a Department of Forestry and Management of the Environment and Natural Resources, Democritus University of Thrace, 193 Pantazidou Street, 68200, Orestiada, Greece; b Research Centre for Tourism, Sustainability and Well-being, University of Algarve, Gambelas Campus, 8005, Faro, Portugal; c Department of Forestry and Natural Resources, Aristotle University of Thessaloniki, Greece

**Keywords:** Tourism, Visitor view, Well-being, Zoological park, Environmental science, Tourism industry, Tourism management, Decision support tools

## Abstract

In an increasingly competitive tourism market, it is essential to assess visitors' demands and levels of satisfaction. Currently, in Greece, there are two public and one private zoo. The Attica zoological park located in Athens has the most extensive collection of animals from all over the world. At the same time, the two public zoos serve a double purpose as zoos and peri-urban parks. A self-administered questionnaire was designed to determine the views and attitudes of the visitors in both public and private zoos of Greece. A total of 707 questionnaires were collected in Attika Park during the weekends of 2017. According to the results, the visitors were mainly middle-aged and highly educated with their motivation for their visits focused on entertainment. They visit the Park mostly in springtime, traveling mainly by car and covering distances of 5–50 km. As regards the quality of infrastructure, facilities, and services available at the zoo, the visitors of Attica Park found access to the area and security provided at the site as very satisfactory. At the same time, they consider that the animal's living conditions, their hygiene, and the existence of shelters for injured animals to be inadequate. The overall satisfaction with the outdoor recreation experience and satisfaction with the existing park facilities and services was higher at the Attica Zoological Park (91.1%) than in the two public zoos of Greece. The results of this work provide lessons that will improve zoo management, animal welfare, and sustain the flow of visitors.

## Teaser

tourism; visitor view; Well-being; zoological park; Environmental Science; Tourism; Tourism Industry; Tourism Management; Decision Support Tools.

## 1 Introduction

Zoos bring people closer to nature through education and by creating environments that immerse visitors into the natural environment. Today, a modern zoo is known as a site of animal conservation, environmental education, research, and especially entertainment (Carr and Cohen, 2011; Ballantyne et al., 2018). Around 10% of the world's population visits a zoo every year (Queiroz and Young, 2018), and according to the World Association of Zoos and Aquariums, more than 600 million people make visits to about 1,200 zoos annually (Holtorf, 2008).

In a competitive tourism market, it is essential to assess visitors’ motivations and levels of satisfaction (Barbeitos et al., 2014). Motivation occurs when an activity satisfies some kind of need (Goossens, 2000). Satisfaction is an essential indicator of tourist experiences while participating in tourism activities (Chi and Qu, 2008), and overall satisfaction is the evaluation based on the overall consumption experience of a good or service. In order to be successful in the market, it is not sufficient to attract new customers, and managers must retain existing customers implementing effective policies of customer satisfaction and loyalty (Dominici and Guzzo, 2010).

Studies of zoo visitors are invaluable for several reasons. They help to inform an understanding of how visitors engage with the zoo, interact with animals, and identify people's needs (Davey, 2007). Some studies focus on physical attributes to measure their overall satisfaction (Pearson et al., 2013) and others to available services (Karanikola et al., 2014). Others focus on hygiene and safety factors provided to visitors, such as toilets and eating facilities, parking, and security services at the site (Jensen, 2007). According to Tomas and Saltmarsh (2012), the assessment of visitors' destinations during their visit can influence the level of their overall satisfaction, while Sickler and Fraser (2009) focus more on visitors' enjoyment than their satisfaction.

Although these studies give us valuable information about visitors’ expectations and satisfaction, the findings differ in different regions, because different cultural values and perceptions may affect the levels of satisfaction. Differences in philosophical orientation concerning animals, nature, and the environment reflect the demands and requirements for satisfaction of zoo visitors; thus, it is essential to meet the values and desires of the society in which it is situated (Dibb, 1995).

Greece has three zoological parks. Two of them are public with free entrance, located at the Thessaloniki peri-urban forest park and inside the city of Trikala in the north and central Greece, respectively. The private zoo called Attica Zoological Park is the largest and located in the Athens metropolitan area, the capital of Greece.

The aim of the present research in Attica zoological park was to form a general opinion of the visitor's demands and levels of satisfaction about recreation, infrastructure, and environmental education. Additionally, a comparison of the views and attitudes of the visitors to public and private zoological gardens may guide zoo managers to understand the level of service and the adequacy of the facilities for visitors and increase zoo attractiveness.

## 2 Literature review

The Zoological Park, in general, is a collection of predominantly wild animals contained in an area of less than 110 ha, which gives people a chance to observe the wildlife that they otherwise might never see (Hunter-Jones and Haywood, 1998). In Greece, the legislation covering zoological gardens is following the European Council Directive 1999/22/EC of 29 March 1999. ‘Zoological park’ means every permanent establishment where wild animals are kept for public exhibition for at least seven days per year regardless of compensation. Circuses and pet shops are exempt from the requirements of zoological parks (Greek law 69Α/3-3-2004).

Zoo visits have many benefits on visitor's well-being because they may have a more accessible approach to wild animals and nature (Fernandez et al., 2009). They find psychological comfort and improve their mental and physical health by enjoying the natural world and interacting with animals (Mitchell and Popham, 2008). The reasons behind people's desire to visit zoos may also be explained by the biophilia hypothesis, which focuses on humans' innate tendency to seek connections with nature and other living organisms (Sakagami and Ohta, 2010; Bruni et al., 2008).

Unfortunately, in modern urban societies, human contact with nature, and especially with the wild fauna, is becoming increasingly rare, particularly for young children (Knight and Herzog, 2009; Karanikola et al., 2012). Zoos seem to be an excellent solution to connect wildlife into the modern world (Williams et al., 2012; Waller et al., 2012; Martens et al., 2019). Additionally, the ongoing biodiversity crisis decreases opportunities to experience nature. However, much research effort has explored the importance of reconnecting people – especially urban dwellers – with nature and conservation issues through experiences of nature (Turley, 1999; Miller et al., 2004).

Although the zoo seems to be a natural place to be away from the daily urban routine, according to Colléony (2016), neither a single nor multiple zoo visits could causally associate to self-reported connectedness to nature. Another point of interest is that many people seek out nature at times of stress (Van den Berg et al., 2010). For example, in 2001, following the attacks on the World Trade Center, managers of national parks observed a pronounced increase in the number of visits. Similarly, zoos in large cities can also provide an outlet for the pressure people are experiencing due to the economic crisis (Karanikola et al., 2014).

Today, despite a shift in emphasis from entertainment towards conservation and education, zoos still need to attract visitors in order to keep operating and ensure economic viability through profitable growth in a competitive market (Linke and Winter, 2010). Since the primary motivation for zoo visits are recreation and contact with nature (Puan and Zakaria, 2007; Sickler and Fraser, 2009), high quality of service is necessary to satisfy the demands and expectations of today's visitors. Zoos are facing challenges because of changes in visitors' expectations for high standards of service. Due to insufficient budgets for improvement, public zoos maintain the traditional management style for exhibits (Lee, 2015).

Zoological parks cannot survive over the long term unless they satisfy the needs of their visitors (Jordaan and du Plessis, 2014; Ogunjinmi et al., 2017). In recent years, public zoos suffered from low finances due to a lack of funding from the local government and the State (Lee, 2015). On the other hand, since the most common source of income for private zoos has traditionally been paying visitors (Hosey et al., 2009). For this reason, zoos must measure visitors’ satisfaction and identify their preferences in order to provide better quality zoo services.

## 3 Material and methods

### 3.1 Study area

**Figure 1:**
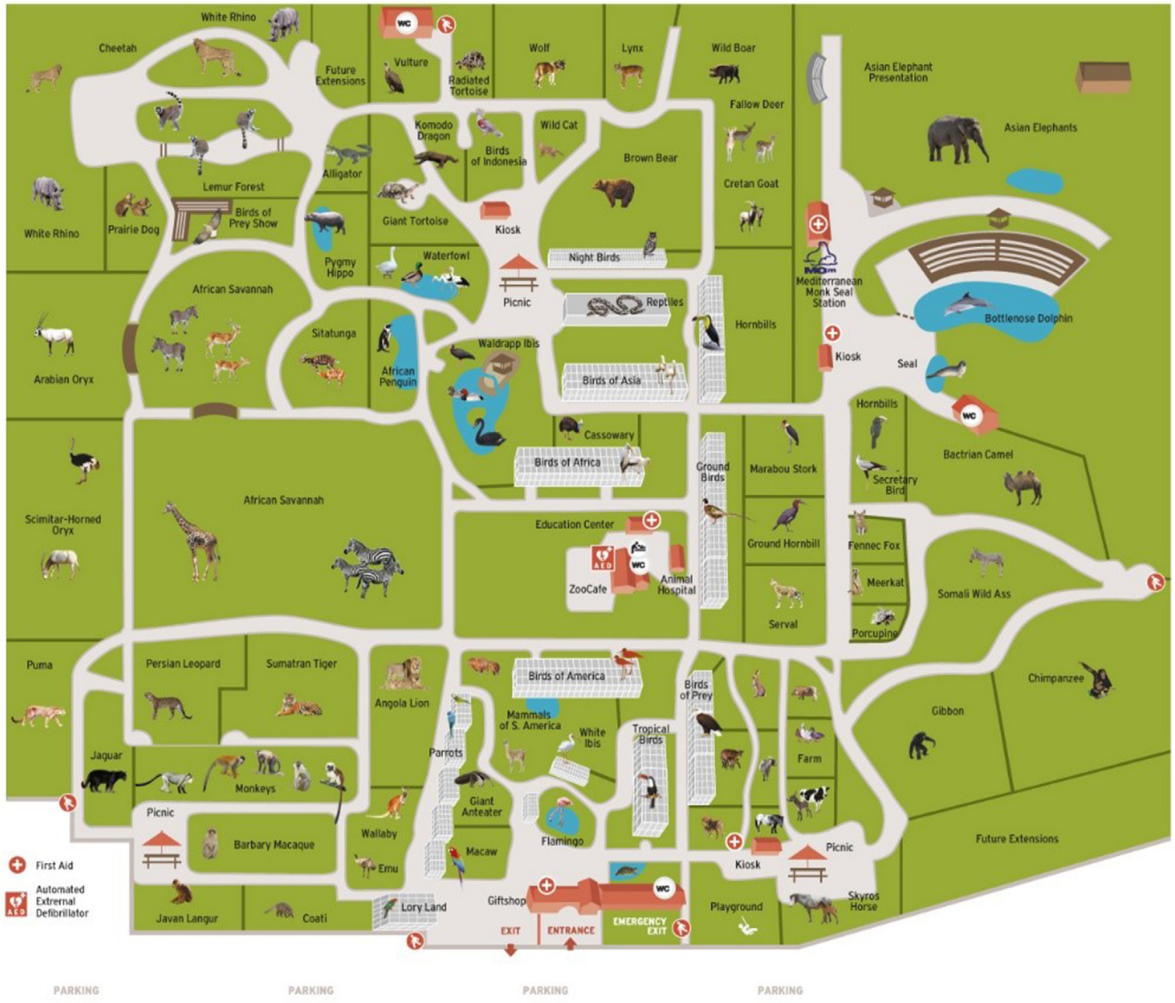
Map of Attica Zoological Park that informs about most animals and zoo sections (Retrieved from http://www.atticapark.com, all rights reserved and used with permission).

The research was conducted at the Attica Zoological Park (latitude 37.981243°N 23.907377°E), located in the municipality of Spata near the city of Athens, Greece. It is a private, 20 ha zoo park, inhabited by approximately 2000 animals belonging to 400 species, with 60% of them classified as threatened species (Figure 1). This zoo established in the year 2000, originally was a bird park that had 300 bird species and a dolphinarium added in 2010. Approximately 380,000 people visit the zoo every year, including 75,000 children following environmental education projects of the zoo.

### 3.2 The survey

This research conducted through face-to-face interviews lasted between 10 to 14 min at the main entrance of the zoo. Regarding the random selection of respondents, the method was skipping five visitors for every person leaving the zoo. Interviews were started at 10:00 am. Additionally, in order to reduce bias, interviews were always conducted by the same person, assuring that explanations and clarifications to the respondents’ doubts were always made the same way.

The questionnaire used in this study had previously conducted at the two public Greek zoos in Thessaloniki and Trikala. The interviewer was stationed at the exit of the Park and approached visitors during the collection period. Participants had to be over 18 years old to respond to the questions of the survey, due to legal requirements in Greece. The survey was divided into three sections: a) general respondent demographics; b) visitors’ views concerning their visit; c) evaluation of the zoo infrastructure and services. The questionnaire consisted of 16 questions that covered a wide range of topics such as frequency, duration, and motivation of the visits, the level of satisfaction with the existing infrastructures; the distance traveled to visit the zoo; and the socioeconomic characteristics of respondents.

The survey included a combination of closed-ended questions and four-point Likert scaled questions, on a scale from 1 (totally inadequate) to 4 (entirely adequate), concerning their satisfaction with the existing services of the zoo (Bigne et al., 2005). Additionally, visitors asked to identify their perceived sense of crowding (Manning, 2007) and to rate the acceptable density of people in the zoo, with results coded in a three-point scale from amused to disturbed (Brace, 2009).

### 3.3 Research method

**Figure 2:**
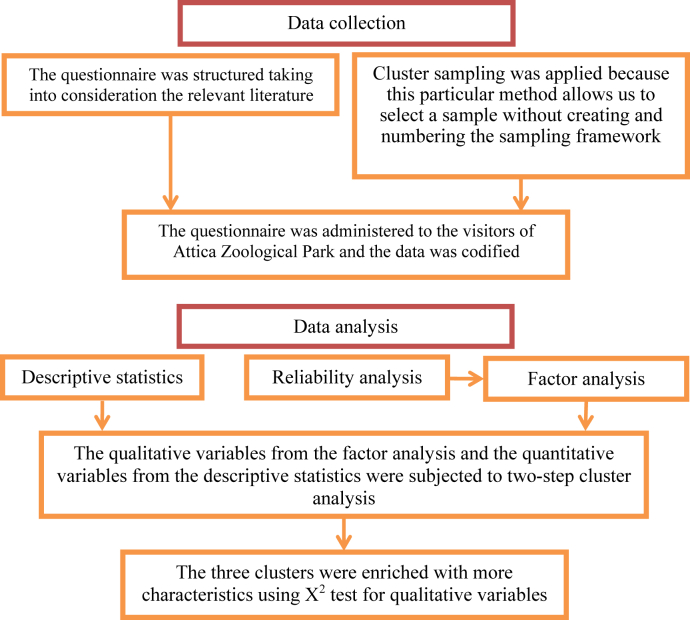
The methodological procedure of the study.

The population under study was the total number of visitors. Figure 2 describes the method procedure. A cluster sampling approach is used because this particular method allows us to select a sample without the necessity to create and number the sampling framework (Kalamatianou, 2000). In cluster sampling, only the existence of one list of groups–clusters are required, along with the elements of the selected clusters (Filias et al., 2000). In our application of this method, the examined clusters were the 52 weekends of the year 2017.

The weekends were chosen as clusters because, during weekends, the zoo visited by the most populational groups (people who work during weekdays and visitors from distant areas that prefer weekends for their visits). We estimated the proportion of the population and the standard error of the proportion of the population s_p_ using the cluster sampling Eqs. (1) and (2) (Arabatzis and Grigoroudis, 2010; Karanikola et al., 2017):

(1) $$p=\frac{\sum_{i=1}^{n}ai}{\sum_{i=1}^{n}mi}$$

(2) $$S_{p}=\sqrt{\frac{N-n}{Nn{\bar{M}}^{2}}\frac{\sum_{i=1}^{n}{\left(ai=pmi\right)}^{2}}{n-1}}$$

where: Ν = the number of clusters in the population; n = the number of clusters in the sampling; m_i_ = the number of population members or sampling units in cluster i (i = 1, 2, 3, …, N); $\bar{m}$ = the average size of clusters in the sample; $\bar{M}$ = the average size of clusters for the population; and α_i_ = the number of the elements in the cluster sampling i that have the specific characteristic of interest.

Pre-sampling was carried out before the final sample, for which five clusters (weekends) were selected. The pre-sampling data were used to estimate the final number of clusters with d = 0.07, with a probability (1-α) = 95%, and the value z_α/2_ = z_0.025_ = 1.96. The maximum sample size was calculated by Eq. (6) to be 14 clusters (weekends). The biggest sample sizes occurred in spring, which was the most important season for visiting because, according to Perkins and Debbage (2016), the ambient thermal environment might influence zoo visitor decisions.

**Table:** 

Cluster	m_i_	a_i_	(a_i_ - p_c_ ∗ m_i_)^2^
1	29	3	11.1111
2	32	2	24.8852
3	36	5	8.1914
4	43	13	13.0263
5	34	15	57.3763
Total	174	38	114.5903

(3) $$p_{c}=\frac{\sum_{i=1}^{n}ai}{\sum_{i=1}^{n}mi}=\frac{38}{174}=0.2184$$

(4) $$s_{c}^{2}=\frac{1}{n-1}\sum_{i=1}^{n}{\left(a_{i}-p_{c}\cdot m_{i}\right)}^{2}=\frac{1}{5-1}114.5903=28.6476$$

Because Μ is unknown, $\bar{M}$ can be estimated as

(5) $$\bar{m}=\frac{1}{n}\sum_{i=1}^{n}m_{i}=\frac{1}{5}174=34.8$$

(6) $$n=\frac{Ns^{2}}{{\left(\frac{d}{z_{\alpha /2}}\right)}^{2}N{\bar{M}}^{2}+s^{2}}=\frac{52\cdot 28.6476}{{\left(\frac{0.07}{1.96}\right)}^{2}\cdot 52\cdot 34.8^{2}+28.6476}=13.6703≅14$$

A total of 707 questionnaires were collected. The response rate was 95%, with 35 visitors refusing to respond because they had no available time. All questionnaires were incorporated into a Microsoft Excel data sheet for data integration. Analyses conducted using the Statistical Package for Social Sciences (SPSS 16.0), including calculating percentages, cross-tabulations, and multivariate statistical technics (Pearson's chi-square).

In the multi-theme variables concerning the factors of the zoo, a reliability analysis was applied. In particular, to find out the internal reliability of a questionnaire (Frangos, 2004), i.e., if our data tended to measure the same thing, we used the α coefficient (or reliability coefficient Cronbach's α). A coefficient α equal or higher to 0.70 is considered satisfactory (Howitt and Gramer, 2003), while higher than 0.80 is considered as very satisfactory. In practice, reliability coefficients with values lower than 0.60 have also been accepted many times (Siardos, 1999).

Factor analysis is used to validate factors common within a group of variables (Sharma, 1996). More specifically, the principal component analysis used here, which based on the spectral analysis of the variance (correlation) matrix. The selection of the number of factors is a dynamic process and presupposes the evaluation of the model in a repeating fashion. In this paper, we used the solution of factors. We also conducted the rotation of the principal components matrix by using the maximum variance rotation method by Kaiser (Djoufras and Karlis, 2001).

Finally, we examined the components that could explain the correlations among the variables of the data. Statistical segmentation of the visitors was undertaken in five distinct groups (clusters) according to the factors of evaluation resulting from the factor analysis (continued variables) and the characteristics of the visitation (categorical variables). A two-step cluster analysis was used for this purpose. This method constitutes a research tool that helps to determine clusters with variables of the same characteristics in a large number of data (questionnaires). The variables were independent of one another; thus, categorical and continued variables handled at the same time following polynomial and the normal distribution, respectively (Harman, 1976; Bacher et al., 2004). Additionally, the correlation of the other variables (continued or categorical) in every cluster separately identified with a check of Pearson's X^2^. In this way, the identity of every cluster is determined with more accuracy.

## 4 Results and discussion

### 4.1 The visitors’ socio-demographic profile

The socio-demographic profile of Attica Park visitors presented in Table 1. The response rate of the surveys was 95% (out of 744 visitors, only 707 completed the interview). The sample included 381 (53.9%) males and 326 (46.1%) females among the 707 respondents. The zoo-visiting public includes groups of all ages, education levels, and diverse social and economic backgrounds, although Martens et al. (2019), in their study in Belgium, reported that the level of concern for animal welfare was distinctly higher among female participants.

**Table 1:** Socio-demographic profile of visitors (s_p_: standard error of proportion).

		p (%)	s_p_
Gender	Male	53.9	0. 0263
	Female	46.1	0. 0226
Age	18–30	38.6	0. 0271
	31–40	40.0	0. 0218
	41–50	16.4	0. 0128
	>50	4.9	0. 0106
Marital status	Unmarried	47.9	0. 0343
	Married	47.1	0. 0282
	Divorced/widowed	4.5	0. 0074
	No answer	0.4	0. 0020
Number of children	Without children	56.7	0. 0320
	One child	19.0	0. 0192
	Two children	18.8	0. 0150
	Three children	4.1	0. 0057
	More than three	1.4	0. 0047
Education level	Primary school	2.3	0. 0058
	Lower secondary	2.1	0. 0020
	Upper secondary	19.5	0. 0116
	Technical school	5.9	0. 0072
	Technological ed	22.3	0. 0244
	University	46.7	0. 0157
	No answer	1.1	0. 0036
Profession	Private employee	36.8	0. 0211
	Public servant	13.9	0. 0215
	Self-employed	17.3	0. 0139
	Farmer	0.7	0. 0020
	Pensioner	2.4	0. 0070
	Student	15.1	0. 0201
	Homemaker	3.5	0. 0068
	Unemployed	10.3	0. 0146
Annual income	≤5.000 €	15.0	0. 0115
	5.001–10.000 €	10.6	0. 0091
	10.001–20.000 €	21.5	0. 0149
	20.001–30.000 €	8.6	0. 0141
	>30.000 €	5.1	0. 0085
	No answer	39.2	0. 0316

In Attica Park, a considerable percentage of the visitors (40%) were within the age of 31–40 years or younger (38.6%), indicating that the weekend visitors were predominantly in their active age. Furthermore, a majority of the visitors were single (47.9%) and highly educated (46.7%), which implies that the public was aware of the importance of zoos and was willing to visit. Also, according to Ogunjinmi et al. (2017), the higher-educated people were more interested in nature recreation activities and were willing to pay to achieve this.

### 4.2 Visitors’ views concerning their visit

**Figure 3:**
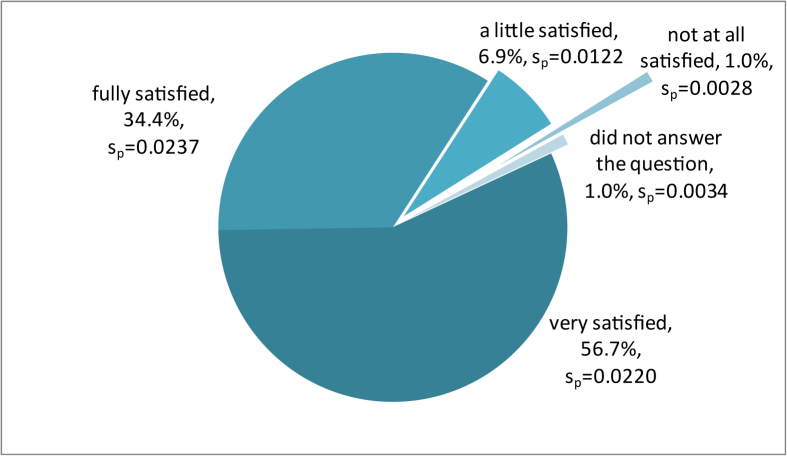
Visitors' overall satisfaction.

To the second section of the questionnaire, visitors asked about the characteristics (satisfaction, frequency, duration, preferable season, motivation, etc.) of their visit. According to this, the visitor's overall satisfaction was very high. The majority of visitors had great overall satisfaction, with 34.4% declaring fully satisfied and 56.7% very satisfied. Only a few of them were little (6.9%) or not at all (1%) satisfied (Figure 3). In the Attica Zoological Park, the satisfaction degree of the visitors is significant. The most common source of revenue for the private zoos has traditionally been the entree fee (Hosey et al., 2009). Visitors are viewed as consumers that are paying for leisure experiences. For this reason, the zoos must measure visitors' satisfaction and identify their preferences in order to provide better quality in zoo services.

Comparing the level of overall satisfaction at the Attica Zoological Park with the two public zoos in Greece, the satisfaction of Attica visitors was the highest. At the zoo in Thessaloniki, most visitors declared little satisfaction (43.5%) to very satisfied (41.1%) with their visit (Karanikola et al., 2014). At the zoo of Trikala, the visitors declared to be little satisfied 41.8% (s_p_ = 0.0216) or very satisfied 38.8% (s_p_ = 0.0418) with their visits (Karanikola et al., 2015).

**Figure 4:**
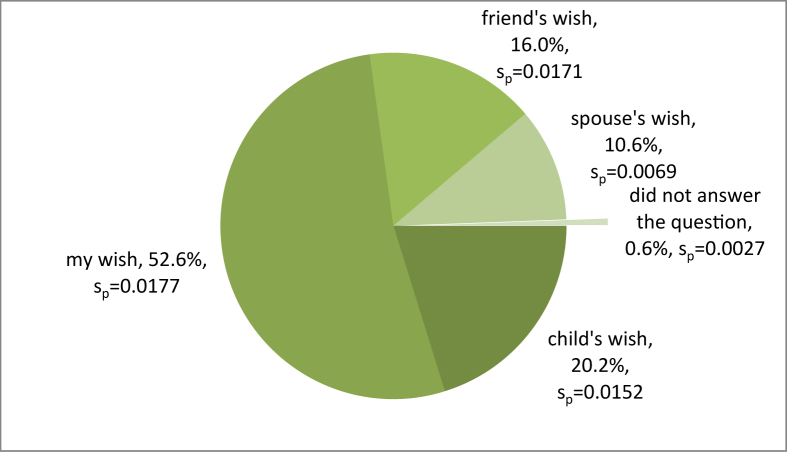
The person with the idea to visit the Attica zoo.

Figure 4 shows that visits to the Attica zoo were primarily motivated by visitors’ own desire (52.6%). In a similar study conducted at the Edinburgh zoo, visitors stated that the main reason for their visit was to go out somewhere with their friends and relatives (Reade and Waran, 1996). Similar studies conducted in Korea by Lee (2015) and in Croatia by Knezevic et al. (2016) listed children as top motivators for zoo visits. Children love to visit zoos and parks, and they urge their parents to take them there on frequent visits.

Crowd management is one of the safety rules put in place in zoos all over the world (Ogunjinmi et al., 2017). Although most of the visitors (59%, s_p_ = 0.0193) in Attica Park were not disturbed by the presence of a crowd, and 24.2% (sp = 0.0118) of them rather enjoyed the crowd presence, 14% (s_p_ = 0.0157) stated that they were disturbed by the crowd. Only a few (0.6%, s_p_ = 0.0022) did not answer the question. This finding is consistent with previous studies at the public zoos of Greece, where 49.1% of the people in Trikala and 57.9% of the people in Thessaloniki do not find inconveniency by the crowd moving around. On the contrary, the presence of people around amused them (40.3% and 33.7%, respectively).

Regarding the frequency of visits to the site, one third (32.5%, s_p_ = 0.0208) answered that they visit the zoo at least once a year, while fewer (8.5%, s_p_ = 0.0073) make even more frequent visits of at least once a month, or once a week (1.3%, s_p_ = 0.0039). The majority (57.4%, s_p_ = 0.0258) of the visitors come to the zoo rarely, while 0.3% (s_p_ = 0.0016) did not answer. The lower frequency of visits in Attica Park compared with that of the Greek public zoos was related to the existing admission fee and the distance the visitors had to travel in order to reach the area.

In similar research conducted in Australia by Ballantyne et al. (2018), the majority of participants reported visiting a zoo at least once per year, while Adetola and Adedire (2018) in Nigeria reported that most of the visitors to the zoo of Ibadan were repeat visitors (78.3%), with more than half of the visitors visiting the zoo more than four times. In the United States, Couch (2013) reported that in Potter Park Zoo, 53% of the visitors were first-time visitors, and Bruni et al. (2008) reported that 38% of the visitors to the Central Park Zoo were there for their first time.

In Attica Zoo, visitation was most sensitive to weather variability. Visitors to Attica Park consider spring and summertime to be the best times of the year for a visit, at a rate of 63.4% (s_p_ = 0.0212) and 14.3% (s_p_ = 0.0124), respectively. Autumn was preferable to 13.8% (s_p_ = 0.0208), and winter to only 5.4% (s_p_ = 0.0066). The question was not answered by 0.1% (sp = 0.0012) of visitors. Hewer and Gough (2016), reported that temperature was the most influential weather variable concerning zoo visitation, followed by precipitation and wind speed.

**Figure 5:**
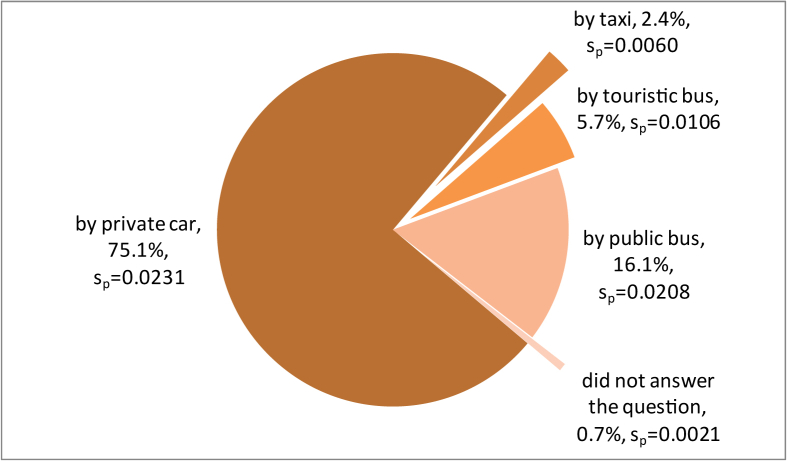
The means of transport used to reach the zoo.

**Figure 6:**
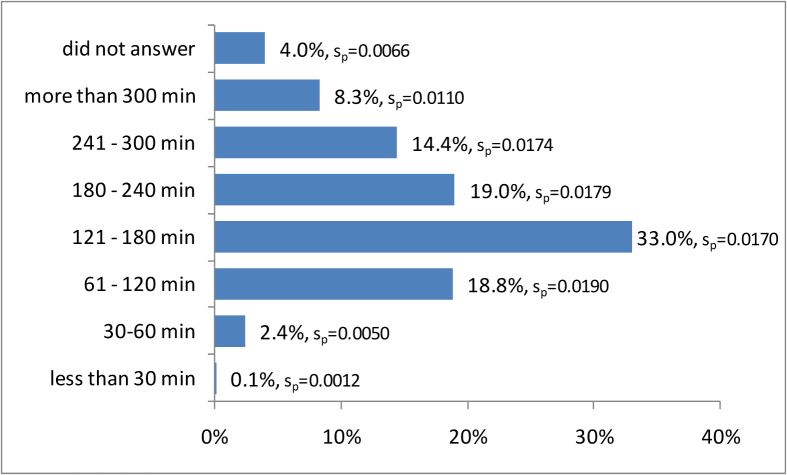
The duration of the visit.

In relation to the distance they travelled to visit the zoo, 3.4% (s_p_ = 0.0059) travelled 0–5 km, 16.1% (s_p_ = 0.0128) travelled 5.1–10 km, 35.9% (s_p_ = 0.0305) travelled 10.1–20 km, and 28.1% (s_p_ = 0.0162) travelled 20.1–50 km, respectively. Finally, 15.4% (s_p_ = 0.0138) of visitors travelled over 50 km, while 1% (s_p_ = 0.0049) did not answer the question. The vast majority reach the site by car (75.1%), and 16.1% use the public bus. Less than 6% use tourist agencies buses, and only 2.4% a taxi (Figure 5). Visits most commonly last from 121–180 min (33.0%), or more than 4 h (19%) (Figure 6). About 8% of the visits last more than 5 h, while only 2.5% are less than 1 h.

### 4.3 Evaluation of the zoo

Visitors asked to evaluate the factors that correlated with the zoo operations and the existing infrastructures and services of the zoo. Although people complain about animal captivity, they still visit zoos and expect to observe animals. Regarding the operation of zoos, the majority, 80.2% (s_p_ = 0.0134) agree with it, while 17.3% (s_p_ = 0.0106) disagree. The question was not answered by 2.5% (s_p_ = 0.0056) of visitors. It should be noted at this point, however, that a large number of those who disagree with the existence of zoos do not visit them as consumers of recreational service. Therefore their views cannot be considered. Similar results were found in public zoo visitors in Greece, with 88.7% and 70.5% of visitors to Trikala and Thessaloniki zoological gardens respectively agreed with zoo operations.

To assess the motivation of visitors’, 61.1% found it adequate for satisfying recreation needs, and 33.8% found it entirely adequate for recreation (Table 2). Concerning acquainting children with animals, 49.6% found the visit adequate, and 45.1% found it entirely adequate. Concerning the provision of environmental education, 53.2% found the visit to be adequate. Also, visitors believe that the zoo is adequate in offering shelter to injured animals, and for the breeding of animals at risk of extinction, at a rate of 57.6% and 53.6%, respectively. Comparing these results with the results from the same questionnaire at the two public Greek zoos, Attica Park scored the highest concerning the recreation, acquainting children with animals, and environmental education. Only the zoo in Attica Park was found to have adequate facilities for sheltering injured animals and for animal breeding.

**Table 2:** Level of importance of visitors' motivation showing the zoo's performance rate.

Factors	Completely Adequate		Adequate		Inadequate		Totally Inadequate		No Answer	
	p (%)	s_p_	p (%)	s_p_	p (%)	s_p_	p (%)	s_p_	p (%)	s_p_
Visitor recreation	33.8	0.0190	61.1	0.0182	4.1	0.0058	0.7	0.0022	0.3	0.0016
Acquainting children with animals	45.1	0.0187	49.6	0.0166	3.7	0.0078	1.1	0.0029	0.4	0.0019
Environmental education	27.2	0.0190	53.2	0.0183	16.5	0.0072	1.6	0.0040	1.6	0.0041
Shelter for injured animals	24.9	0.0188	57.6	0.0092	11.2	0.0140	1.4	0.0031	5.0	0.0110
Animal breeding	24.8	0.0163	53.6	0.0127	15.7	0.0121	1.6	0.0032	4.4	0.0039

Many zoo studies have explored visitors' motivation. They show that zoos, in general, give people a chance to view wildlife they may otherwise never see (Carr and Cohen, 2011; Waller et al., 2012). According to investigations by Adetola and Adedire (2018), the primary motivations for visiting Southwest Nigeria's zoos were to see the conditions of habitat and diversity of wildlife, zoo proximity to their residence, and viewing the wild animals. Jordaan and du Plessis (2014) found that people visit the Zoological Garden in South Africa to have a self-directed zoo experience for recreation and relaxation. Conway (2003) considers that zoos must become proactive conservation organizations, applying their popularity to win support for wildlife protection and their expertise to help sustain reduced numbers of wildlife in marginal habitats. However, according to Smith et al. (2008), in the future, zoos might be able to become centers of conservation rather than living museums, and for this to happen, significant changes to the layout would be required (Ryan and Saward, 2004).

Zoo management must carefully balance the need for funding and the demands of visitors with the needs of individual animals and species. Reconciling the demands on income is a difficult task. That is most obvious in the context of providing visitor facilities, which is an investment that generally comes at the expense of the animals (Turley, 1999). Table 3 shows that visitors find access to zoo ease, and the existence of a parking area to be excellent (54.1%) and good (41.9%). Similarly, as regards the overall area occupied by the zoo, for 32.8% of the visitors, it is entirely adequate, while 55.4% say it is adequate. Regarding the landscaping of the site, 65.3% state they were delighted, while 10.5% say they were minimally satisfied with it.

**Table 3:** Evaluation of zoo infrastructures (physical attributes and services).

	p (%)	s_p_	p (%)	s_p_	p (%)	s_p_	p (%)	s_p_	p (%)	s_p_
Ease of access and existence of a parking area	Excellent		Good		Bad		Very Bad		No Answer	
	54.0	0.0244	41.9	0.0203	3.0	0.0068	0.7	0.0028	0.4	0.0026
Area covered by the zoo	Completely Adequate		Adequate		Inadequate		Highly Inadequate		No Answer	
	38.2	0.0207	55.4	0.0159	5.5	0.0077	0.7	0.0023	0.1	0.0012
Landscaping of the site	Fully Satisfied		Very Satisfied		Minimally Satisfied		Not at all Satisfied		No Answer	
	22.8	0.0152	65.3	0.0137	10.5	0.0141	0.8	0.0030	0.6	0.0027
Available infrastructure (kiosks, sits e.tc.)	Very Good		Good		Bad		Very Bad		No Answer	
	37.5	0.0185	58.1	0.0148	3.7	0.0057	0.7	0.0028	0.0	
Services provided to visitors	Very Good		Good		Bad		Very Bad		No Answer	
	36.9	0.0168	55.7	0.0175	6.2	0.0136	0.7	0.0028	0.4	0.0019
Security at the site, particularly for children	Very Good		Good		Bad		Very Bad		No answer	
	41.4	0.0219	53.7	0.0205	3.5	0.0066	0.8	0.0028	0.4	0.0019
Abundance of animals	Very High		High		Low		Very Low		No Answer	
	24.2	0.0165	62.4	0.0203	10.9	0.0113	2.3	0.0067	0.3	0.0016
Variety of plants	Very Large		Large		Small		Very Small		No Answer	
	9.5	0.0154	55.6	0.0179	30.1	0.0171	4.5	0.0091	0.3	0.0016
Animal enclosures	Completely Adequate		Adequate		Inadequate		Highly Inadequate		No Answer	
	12.6	0.0161	63.9	0.0150	20.8	0.0133	1.8	0.0036	0.8	0.0031
Living conditions of the animals	Fully Satisfied.		Very Satisfied.		Minimally Satisfied		Not at all Satisfied		No Answer	
	12.7	0.0150	67.2	0.0166	17.4	0.0114	1.3	0.0020	1.4	0.0032
Hygiene and safety conditions for the animals	Fully Satisfied		Very Satisfied		Minimally Satisfied		Not at all Satisfied		No Answer	
	17.5	0.0133	67.3	0.0140	12.6	0.0100	1.0	0.0020	1.6	0.0043
Friendliness of the staff	Very Good		Good		Bad		Very bad		No Answer	
	48.2	0.0211	47.8	0.0135	2.3	0.0071	0.8	0.0027	0.8	0.0038

Concerning the infrastructure available at the zoo (kiosks, toilets), 58.1% of visitors consider it to be good, and 37.5% excellent. As regards the services provided to visitors (information, cleanliness), 55.7% of visitors believe they were right and 36.9% find them very good. They also evaluated the security aspects of the site, particularly for children, with 53.7% describing them as good and 41.4% as very good. According to the friendliness of the staff, the visitors were delighted and characterized it as very good (48.2%) and good (47.8%).

Regarding the abundance of animals at the zoo, 62.4% and 24.2% of visitors believe it is high and very high, respectively. A majority of visitors found the zoo's variety of plants to be high (55.6%), while 30.1% found the variety small.

Regarding the extent to which the animals' enclosures represent their natural environment, 63.9% of visitors believe they were adequate, while 20.8% find them inadequate. Furthermore, regarding the animals' living conditions, 67.2% consider them to be very satisfactory, and 17.4% less satisfactory. Visitors evaluate the animals’ hygiene and safety conditions, with 67.3% considering them to be very clean and safe, and 17.5% absolutely satisfied.

Table 3 shows the visitors' highly evaluated factors that correlated with the zoo infrastructure, visitors' safety and accessibility, the landscape design, and the service's attributes. Plant and animal variety, animal enclosures, and their living conditions, and safety and hygiene were rated lower. Comparing the results presented in Table 2 with the study at the public zoo of Thessaloniki, we verified that the level of satisfaction with the infrastructures at Attica Park was better, except in plant variety, which the zoo of Thessaloniki surpasses (Karanikola et al., 2014).

According to Lee (2015), the essential attributes identified by Korean visitors after applying factor analysis were visitors’ safety, ease of viewing, and child-friendly displays, which were about the same as the present study. Meanwhile, in similar research conducted by Ogunjinmi et al. (2017) in Nigeria, most of the visitors (57%) were aware of the safety rules and guidelines of the zoo, which shows a high level of safety literacy amongst visitors.

Reliability analysis applied for the above multi-theme variable. The reliability coefficient α was 0.875, and after the application of factor analysis, extracted four factors. The first factor we named “infrastructures and services for the visitors”; the second, “infrastructures and services for the animals”; the third, “basic planning of the zoo”; and the fourth we named “animal and plant abundance” (Table 4). In similar research conducted by Lee (2015) in the zoos of Korea, the five factors excluded from the factor analysis were not precisely the same: visitor's safety, the well-being of the animals, zoo's infrastructure, professional guides, and educational programs.

**Table 4:** Factor analysis loadings after rotation (bold numbers show the choosen factor belongs to every variable).

Variables	Factors loadings			
	1	2	3	4
Ease of access and existence of a parking area	0.328	0.036	**0.733**	-0.028
Area covered by the zoo	0.065	0.284	**0.772**	0.217
Landscaping of the site	0.249	0.321	**0.671**	0.154
Available infrastructure (kiosks, benches, etc.)	**0.611**	0.242	0.332	0.176
Services provided to visitors	**0.792**	0.208	0.195	0.137
Security at the site, particularly for children	**0.711**	0.196	0.222	0.056
Abundance of animals	0.106	0.106	0.103	**0.861**
Variety of plants	0.204	0.178	0.118	**0.800**
Animal enclosures	0.240	**0.773**	0.204	0.112
Living conditions of the animals	0.236	**0.853**	0.198	0.136
Hygiene and safety conditions for the animals	0.269	**0.822**	0.176	0.156
Friendliness of the staff	**0.709**	0.200	0.060	0.145

The number of clusters was determined by applying the two-step cluster analysis in SPSS. The observations were grouped into three clusters as the optimum solution. More specifically, of the 707 respondents, 57% were placed in the first cluster, 8% in the second cluster, and 35% in the third cluster.

**Figure 7:**
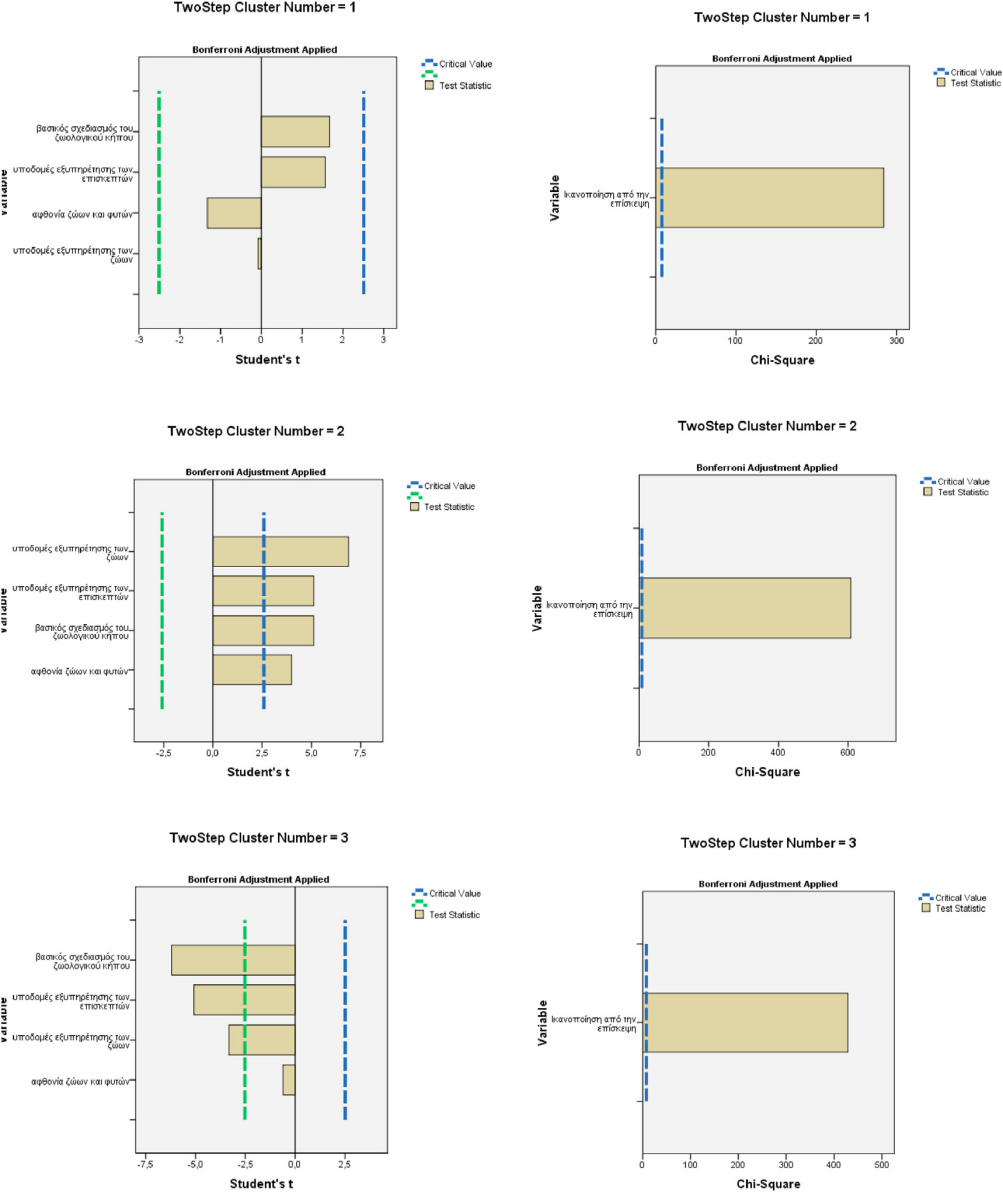
Diagrammatic representations of statistical tests of variables per cluster (a, c and e continuous; and b, d, and f categorical variables).

According to the diagrammatic representations of statistical tests for the four continuous variables, we observed that all of them tended to play a significant role in the formation of the second cluster (Figure 7a, c, e). For the categorical variables, the value of the statistical X^2^ of the one variable to the three clusters exceeded the limits of the critical value (Figure 7b, d, f). The characteristics of the clusters are presented in Table 5. From the check of Pearson's X^2^, among other characteristics of the visit, the three clusters observed statistical significance (α < 0,001 και α < 0,05). Table 5 shows the relation between them.

**Table 5:** Interpretation of the clusters’ observations.

Variables	Cluster 1	Cluster 2	Cluster 3
Infrastructure and services for the visitors	Moderate	Negative	Positive
Infrastructure and services for the animals	Moderate	Negative	Positive
Basic planning of the zoo	Moderate	Negative	Positive
Abundance of animals and plants	Moderate	Negative	Moderate
Satisfaction from the visit	Very Satisfied	Little or not at all Satisfied	Absolutely Satisfied
Wish of whom ∗	Friends or Spouse	Friends or Spouse	Mine or children
Frequency of visit ∗∗	Once or more/week	Rarely	Once/year
Distant cover for the visit ∗∗	10.1–20 km	More than 20 km	Less than 10 km 20.1–50 km
Means of transport	Mainly by Car	Touristic Bus	Public Bus
Accept zoo operation ∗	Yes	No	Yes
Visitor's Recreation ∗	Adequate	Inadequate to Totally Inadequate	Completely Adequate
Acquainting children with animals ∗	Adequate	Inadequate to Totally Inadequate	Completely Adequate
Environmental education∗	Adequate	Inadequate to Totally Inadequate	Completely Adequate
Shelter for injured animals∗	Adequate	Inadequate to Totally Inadequate	Completely Adequate
Animal breeding ∗	Adequate	Inadequate to Totally Inadequate	Completely Adequate
Crowding in the zoo ∗	Paid no attention	Disturbed by them	Amused by them

Statistical significance α < 0.001, ∗∗α < 0.05.

After applying the two-step cluster analysis, observations were grouped into three clusters of visitors with different characteristics. The first cluster comprises the majority (57%) of visitors with high satisfaction with their visit but mediocre opinions related to infrastructure services for both visitors and animals, for zoo planning, and about animals and plant abundance. Their visit was motivated by family or friends’ wishes, and they traveled distances up to 20 km, mainly by car. They visit the zoo at least once a week or once a month, and their perceived sense of crowding made no difference to them. The visitors in the first cluster accept zoo operations, and they believed that Attica Park is adequate to satisfy the desire for recreation, acquainting children with animals and environmental education. Also, first-cluster visitors found sheltering of injured animals and for animal breeding to be adequate.

The second cluster comprised a minority 8% of the visitors that declared low satisfaction with their visit and provided negative opinions regarding the infrastructure, services, planning of the zoo, and diversity of plants and animals. They come to the zoo rarely, by tour buses and travel distances longer than 20 km. They declared that the crowd disturbed them; they had negative opinions about zoo operation and found that Attica Park provided inadequate shelter for injured animals or animals that were breeding. They also found the Park inadequate for supplying recreation, acquainting children with animals, and providing environmental education.

The third cluster is characterized by higher visitor satisfaction. They were positive about all infrastructure services for the animals and visitors and evaluated the abundance of animals and plants as mediocre. They come to the zoo mainly by public bus, and they found the crowd around them amusing. They thought that Attica Park provided the best way to recreate and connect with nature through environmental education and acquainting children with animals.

## 5 Conclusions and recommendations

The profile of visitors and their motivations to visit Attica zoo were examined in order to assist Park management. Our study about Attica Park found that the majority of the visitors were middle-aged or young and highly educated. They visit Attica Park mostly in springtime, traveling mainly by car and covering distances of 5–50 km. Their primary motivations focused on entertainment, acquainting children with animals, and enhancing their connection with nature. In general, the vast majority of the visitors agree with the operation of the zoo. However, they consider the existence of shelters for injured animals to be inadequate, as well as how animals at risk of extinction bred.

As regards the quality of infrastructure and available facilities at the zoo, the following responses noted: access to the area is secure, and the parking area found to be very satisfactory. The overall area covered by the zoo is adequate, and the landscaping of the site is considered to be satisfactory. Furthermore, regarding the services provided to visitors and the security provided at the site, respondents stated to a great extent that they were outstanding. According to the majority of visitors, animals were abundant, in contrast to the low variety of plants. Also, visitors had the opinion that the animal's living conditions, as well as their hygiene and safety conditions, were adequate.

The application of a two-step cluster analysis revealed three clusters of visitors with different characteristics that administrators should take into consideration for the sustainable management of the Park.

The first cluster included frequent zoo visitors generally satisfied with the experience in the zoo, but still searching for new challenges and activities. Zoo managers could satisfy those needs by innovating ways to deliver information about animal life. For example, visitors could get an invitation to feed birds of prey or watch the birth of a mammal. They could enjoy a bird’ s-eye view using a drone that could fly over the less approachable areas of the zoo. Also, additional changes to the information technology may improve the image of the zoo (for example, information about the resident animals and plants sent to the mobile phones of the visitors).

For the second cluster, with the least satisfied visitors, park managers need innovative approaches to change their negative opinion of the zoos. For example, the application of digital marketing tools and augmented reality apps that will exhibit the role of the zoo that show videos with related themes that enhance the experience. An example would be the settlement of an enclosure with an injured falcon until its recovery. These steps would also improve the image of the zoo, highlighting its role as a park that protects the natural populations of endangered species.

For the third cluster, which included visitors with the highest satisfaction, we propose to attract more of those visitors and give them gifts (for example, free bus tickets or family discounts) to increase the frequency of their visits. We recommend to offer them opportunities to share their experiences during their visit with their social network and share augmented reality memories. The above may attract more visitors, especially from the younger groups of society.

Comparing this private zoo with the two public zoos in Greece, it was revealed that the elderly and parents accompanied by their children had a much lower presence in the private zoo. An explanation for this low presence may be the relatively high admission price that, in the time of economic crisis, leads families to seek cheaper modes of family entertainment. This was also the reason that the frequency of the Attica Zoological Park visits was lower than at the public Greek zoos, while the duration was longer. Families and friends visit Attica Park only when they have plenty of time because of the admission fee cost, while for short time park visits for recreation, they go to closer urban green parks.

Overall satisfaction at the Attica Zoological Park was higher than the two public zoos of Greece. Even though all three zoos were highly evaluated for recreation, acquainting children with animals, and environmental education, only the Attica Park zoo was evaluated as having adequate facilities for sheltering injured animals or for animal breeding. A comparison of public and private zoological gardens visitors' views and attitudes may guide zoo administrators to optimize zoo space management, directing attention towards more effective utilization of scarce resources and increase the zoo attractiveness and animals’ welfare. The recent increase of public interest in the role of Greek zoos as educational and conservation centers is expected to encourage more research on the demands of zoo visitors and their satisfaction.

Limitations to the research constitute the findings based on comparison from data of the three zoological parks of Greece. Although the questionnaire was the same, the survey was conducted at different times, so the data could not incorporate the same figures and Tables. The survey should be conducted in the same year to avoid the influence of the recent economic crises and to generalize the findings for visitors from all Greek zoos.

## Declarations

### Author contribution statement

T. Panagopoulos: W Analyzed and interpreted the data; Contributed reagents, materials, analysis tools or data; Wrote the paper.

P. Karanikola: Conceived and designed the experiments; Performed the experiments; Wrote the paper.

S. Tampakis: Conceived and designed the experiments; Analyzed and interpreted the data.

A. Tampakis: Performed the experiments.

### Funding statement

This research did not receive any specific grant from funding agencies in the public, commercial, or not-for-profit sectors.

### Competing interest statement

The authors declare no conflict of interest.

### Additional information

No additional information is available for this paper.

## Appendix A Supplementary data

English_zoo_survey English_zoo_survey
