# Corrigendum to “Visitor preferences and satisfaction in Attica zoological park, Greece” [Heliyon 9 (9) (September 2020) e04935]

**DOI:** 10.1016/j.heliyon.2022.e09392

**Published:** 2022-05-12

**Authors:** Paraskevi Karanikola, Thomas Panagopoulos, Stilianos Tampakis, Antonios Tampakis

**Affiliations:** a Department of Forestry and Management of the Environment and Natural Resources, Democritus University of Thrace, 193 Pantazidou Street, 68200, Orestiada, Greece; b Research Centre for Tourism, Sustainability and Well-being, University of Algarve, Gambelas Campus, 8005, Faro, Portugal; c Department of Forestry and Natural Resources, Aristotle University of Thessaloniki, Greece

In the original published version of this article, there were 2 minor typos in the Abstract and Conclusion sections, which reversed the intended meaning of these statements. In the Abstract, the sentence "At the same time, they consider that the animal's living conditions, their hygiene, and the existence of shelters for injured animals to be inadequate” has been corrected to "At the same time, they consider that the animal's living conditions, their hygiene, and the existence of shelters for injured animals to be adequate."

In the Conclusion, the statement "However, they consider the existence of shelters for injured animals to be inadequate" has been corrected to "However, they consider the existence of shelters for injured animals to be adequate." The authors apologize for these errors. Both the HTML and PDF versions of the article have been updated to correct the errors.

