# Supplementary material for: Visitor preferences and satisfaction in Attica zoological park, Greece
Source: Heliyon. 2020 Sep 14;6(9):e04935. doi: 10.1016/j.heliyon.2020.e04935 (PMC7498856; doi:10.1016/j.heliyon.2020.e04935)
Supplement: English_zoo_survey [file mmc1.doc]

#
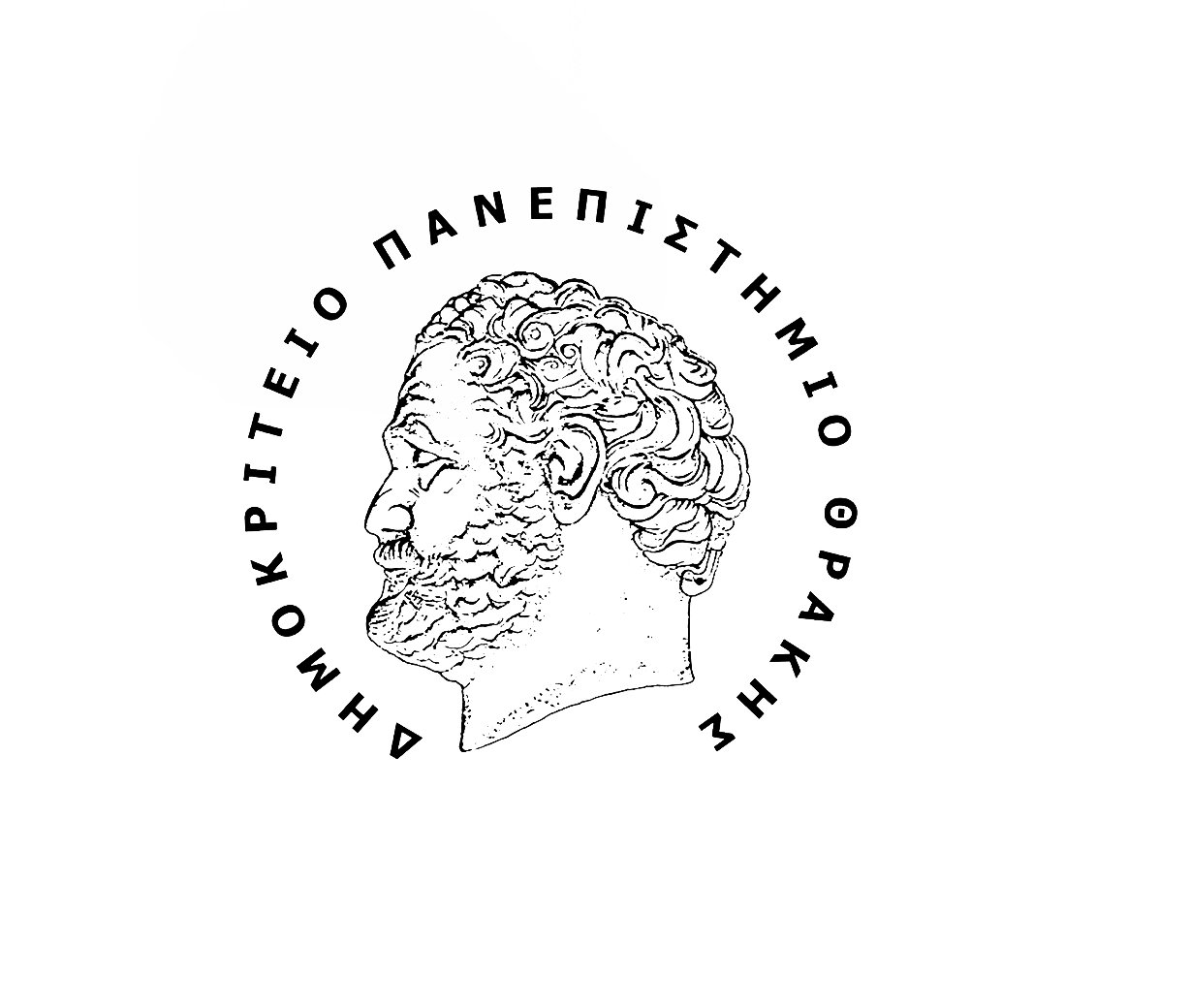
DEMOCRITUS UNIVERSITY OF THRACE

**DEPARTMENT OF FORESTRY AND MANAGEMENT OF THE ENVIRONMENT AND NATURAL RESOURCES**

# Dear visitor

This questionnaire is a tool for academic research about the perceptions of visitors in Attica Zoological Park. The information you give is anonymous and will use it only for the research. Please answer the questions below with attention and honesty.

## **QUESTIONNAIRE**

1. **How satisfied are you from your visit to Attica Zoological Park?**

Absolutely satisfied **** Very satisfied **** Little satisfied **** Not at all satisfied. ****

1. **What distance did you cover to reach** **Attica Zoological Park?**

0 - 5 Km **** 5,1 - 10 Km **** 10,1 - 20 Km **** 20,1 - 50 Km **** > 50 Km ****

**3. What means of transportation you use to come to Zoological Park?**

Private car**** public bus **** touristic bus **** taxi ****

1. **The visit was motivated by…**

My own desire **** Friends **** Spouse **** children ****

1. **How often you visit Attica Zoological Park?**

✍ ……… times/week ✍ ……… times/month

✍ ……… times/year rarely …………………. ✍

6. How much time your visit last: ……… ✍

**7. What is the ideal season to visit the park?**

fall  winter  spring  summer 

8. During your visit to the park the people around:

Disturb you **** Amuse you ****

Pay no attention **** Something else…..…………. ✍

**9. Do you agree with the operation of Zoological parks and gardens?**

Yes  No 

**10. In what degree the zoo respond to…**

**Visitor’s amusement**

Absolutely sufficient **** Sufficient **** Insufficient  Absolutely insufficient 

**Acquaintance of children with animals and nature**

Absolutely sufficient  Sufficient  Insufficient  Absolutely insufficient 

**Environmental education of people**

Absolutely sufficient  Sufficient  Insufficient  Absolutely insufficient 

**Shelter of injured animals**

Absolutely sufficient  Sufficient  Insufficient  Absolutely insufficient 

#### Breeding of animals in danger

Absolutely sufficient  Sufficient  Insufficient  Absolutely insufficient ****

11. Evaluate the existing situation of the zoo:

**Convenience in access and parking existence**

Very good  Good  Bad  Very bad 

#### Total area of the zoo

Absolutely sufficient  Sufficient  Insufficient  Absolutely insufficient ****

**Architectural design of the place**

#### Absolutely sufficient  Sufficient  Insufficient  Absolutely insufficient 

####

#### Available infrastructures (kiosks, toilets, sits, e.tc.)

#### Very good  Good  Bad  Very bad 

#### Available services for the visitors (information, cleanliness)

Very good  Good  Bad  Very bad 

**Security at the Site, Particularly for Children**

#### Very good  Good  Bad  Very bad 

#### Abundance of Animals

Very low  Low High  Very high 

#### Variety of Plants

Very large  Large  Small  Very small 

**Animal Enclosures**

#### Absolutely sufficient  Sufficient  Insufficient  Absolutely insufficient 

####

**Hygiene and Safety Conditions for the Animals**

Absolutely satisfied  satisfied  Minimally satisfied  Not at all satisfied 

####

**friendliness of the staff**

#### Very good  Good  Bad  Very bad 

**13. Gender:** Man  Woman ****

**14.** Age**:** 18-30  31 – 40  41 – 50  > 50 

**15. Education:** Primary School **** Lower Secondary School **** Upper Secondary School **** Technical School **** Technical Education **** University****

**16. Marital status:** Single  Married  Number of children**:** ……… ✍

**17) Profession:** Private employee  Farmer- Stock keeper  Worker

Public servant  Student Housewife

Self-employed Pensioner  Unemployed 

**18) Annual income:**

Less than 5.000 €  5.001 - 10.000 €  10.001 - 20.000 € 

20.001 – 30.000 €  More than 30.000 €  Not answer 
